# Quality appraisal of clinical practice guidelines for the evaluation and management of primary hyperparathyroidism

**DOI:** 10.1007/s12020-024-03790-8

**Published:** 2024-03-29

**Authors:** Mandy K. Salmon, Christian G. Fritz, Louis-Xavier Barrette, Dominic Romeo, Neeraj V. Suresh, Jinggang J. Ng, Eesha Balar, Aman Prasad, Alvaro Moreira, Karthik Rajasekaran

**Affiliations:** 1 Department of Otorhinolaryngology-Head and Neck Surgery, University of Pennsylvania, Perelman School of Medicine, Philadelphia, PA USA; 2 Department of Otolaryngology-Head & Neck Surgery, Oregon Health & Science University, Portland, OR USA; 3 Division of Otolaryngology-Head and Neck Surgery, Department of Surgery, Yale University School of Medicine, New Haven, CT USA; 4 Department of Pediatrics, University of Texas Health Science Center-San Antonio, San Antonio, TX USA; 5 Leonard Davis Institute of Health Economics, University of Pennsylvania, Philadelphia, PA USA

**Keywords:** Primary hyperparathyroidism, AGREE II, consensus statement, clinical practice guidelines, management

## Abstract

**Purpose:**

Multiple groups have created clinical practice guidelines (CPGs) for the management of primary hyperparathyroidism (PHPT). This report provides a rigorous quality assessment using the Appraisal of Guidelines for Research & Evaluation Instrument (AGREE II) to identify high-performing guidelines and areas for improvement.

**Methods:**

A systematic review was conducted to isolate CPGs addressing the management of PHPT. Guideline data was extracted and quality ratings were assigned by four independent reviewers. Intraclass correlation coefficients (ICC) were calculated to ensure interrater reliability.

**Results:**

Twelve guidelines were assessed. The American Association of Endocrine Surgeons (AAES) guideline had the highest mean scaled score across all domains (73.6 ± 31.4%). No other published guideline achieved a “high” quality designation. The highest scoring domain was “clarity of presentation” (mean 60.5 ± 26.5%). The lowest scoring domain was “applicability” (mean 19.8 ± 18.2%). Scoring reliability was excellent, with ICC ≥ 0.89 for all AGREE II 6 domains.

**Conclusion:**

Although several working groups have developed guidelines to address PHPT management, only those published by the AAES meet all methodologic quality criteria necessary to ensure incorporation of recommendations into clinical practice. Future guidelines would benefit from the development of tools, resources, monitoring criteria that enhance applicability.

## Introduction

Primary hyperparathyroidism (PHPT) is an endocrine disorder caused by either parathyroid adenoma, parathyroid hyperplasia, or parathyroid carcinoma [1]. The disorder is relatively common, with an incidence of 66 per 100,000 person-years in females and 25 per 100,000 person-years in males [2]. PHPT can affect many organ systems, resulting in renal disease, osteoporosis, hypertension, depression and constipation [3]. Currently, only 20–30% of patients in developed countries present with symptoms, while the majority of asymptomatic patients are diagnosed by lab abnormalities [4, 5]. Approximately one in four of these asymptomatic patients develop symptoms within five years, underscoring the importance of prompt diagnosis and management [6].

PHPT can present in many forms. The classic presentation involves elevations in both calcium and parathyroid hormone. Variants such as normo-calcemic hyperparathyroidism and hypercalcemia with inappropriately normal parathyroid hormone are common as well, with some studies suggesting these variants affect approximately 16% and 7% of patients presenting for evaluation of PHPT, respectively [7, 8]. Surgery is currently the definitive treatment of choice for preventing long-term complications of PHPT [9]. For patients who are not surgical candidates, pharmacological management may be undertaken to address symptoms such as osteoporosis by utilizing bisphosphonates to improve bone mineral density and cinacalcet to improve serum calcium levels [10, 11]. The landscape of PHPT diagnosis has changed dramatically over the last few decades, with progression from ultrasound and sestamibi scans to evaluation with four-dimensional CT imaging and (11)C-choline or (18)F-choline PET/CT [12].

The aforementioned technological advances have influenced guidelines addressing the diagnosis and management of PHTP. Despite the recent updates, many questions remain concerning the appropriate mechanisms for intervention, especially in patients who are asymptomatic or unable to undergo surgery. Additionally, the heterogeneity of presentation, with some patients presenting with normocalcemia or normal parathyroid hormone levels, further complicates the decision to intervene surgically or continue monitoring with close follow-up. Management of PHPT includes multidisciplinary efforts from endocrinologists, endocrine surgeons, and otolaryngologists. To standardize the criteria for surgical intervention and to address these concerns, multiple professional societies and organizations have developed consensus documents to guide management of PHPT [13–25]. Guideline quality assessment is now possible using The Appraisal of Guidelines for Research & Evaluation Instrument (AGREE II) [23]. The purpose of this study is to assess the quality of guidelines addressing management and evaluation of PHPT.

## Materials and methods

A systematic search of the literature was performed in April 2023 using SCOPUS, PubMed, and EMBASE for the following terms: [(“primary hyperparathyroidism”) AND (“guideline” OR “consensus” OR “recommendation” OR “clinical practice guideline”)]. Duplicates were removed, as well as any articles not written in the English language. All articles were then reviewed by title and article type. Reviews, randomized control trials, textbook chapters, and case reports were excluded. The remaining guidelines underwent full abstract review and those addressing management of PHPT were selected for inclusion. This retrospective review of previously published guidelines was deemed exempt from review (UPenn-2023-00018.1) by the University of Pennsylvania Health System Institutional Review Board.

### Data collection

Following the systematic search, general information about each included guideline was collected: authorship, publication year, funding source, development body, evidence base, and guideline content. Additionally, the most important clinical pearls were summarized for each guideline.

### Appraisal and scoring

Each included guideline was appraised using the AGREE II instrument. Four reviewers (EB, DR, AP, NS) received a certificate of competency upon completion of training that encompassed an in-depth interrogation of the 23 sub-domains of the AGREE II instrument [26]. These reviewers independently assessed and rated each included article. Data was aggregated, quantitively scaled, and assessed according to each of the six major domains of the AGREE II instrument: scope and purpose, stakeholder involvement, rigor of development, clarity of presentation, applicability, and editorial independence. These domains and 23 sub-domains are summarized in Table 1.

**Table 1:** Summary of the domains and sub-domains evaluated with the AGREE II instrument

Scope and purpose	
1	The overall objective(s) of the guideline is (are) specifically described.
2	The health question(s) covered by the guideline is (are) specifically described.
3	The population (e.g., patients, public) to whom the guideline is meant to apply is specifically described.
Stakeholder involvement	
4	The guideline development group includes individuals from all relevant professional groups.
5	The views and preferences of the target population (e.g., patients, public) have been sought.
6	The target users of the guideline are clearly defined.
Rigor of development	
7	Systematic methods were used to search for evidence.
8	The criteria for selecting the evidence are clearly described.
9	The strengths and limitations of the body of evidence are clearly described.
10	The methods for formulating the recommendations are clearly described.
11	The health benefits, side effects and risks have been considered in formulating the recommendations.
12	There is an explicit link between the recommendations and the supporting evidence.
13	The guideline has been externally reviewed by experts before its publication.
14	A procedure for updating the guideline is provided.
Clarity of presentation	
15	The recommendations are specific and unambiguous.
16	The different options for management of the condition or health issue are clearly presented.
17	Key recommendations are easily identifiable.
Applicability	
18	The guideline describes facilitators and barriers to its application.
19	The guideline provides advice and/or tools on how the recommendations can be put into practice.
20	The potential resource implications of applying the recommendations have been considered.
21	The guideline presents monitoring and/or auditing criteria.
Editorial independence	
22	The views of the funding body have not influenced the content of the guideline.
23	Competing interests of guideline development group members have been recorded and addressed.

*AGREE II* Appraisal of Guidelines for Research and Evaluation Instrument

Scores within each of the domains were converted into scaled domain scores according to the standardized formula validated by AGREE-II trust:

$$\displaystyle {scaled\,domain\,score} = \dfrac{{obtained\,domain\,score - minimum\,domain\,score}}{{maxmimum\,domain\,score - minimum\,domain\,score}}x100{{{\mathrm{\% }}}}$$

The resultant value arising from this scaled domain score formula was used to assign quality rankings. Guidelines with scaled domain scores of >60% in ≥5 domains were defined as “high” quality, in accordance with the standardized rating protocol (https://www.agreetrust.org/resource-centre/agree-ii/faq-agree-ii-2/#:~:text=Answer%3A%20The%20AGREE%20II%20has,with%20sufficient%20inter%2Drater%20reliability.). Guidelines with scaled domain scores of >60% in 3–4 domains were defined as “average” quality, and CPGs with scores of >60% in ≤2 domains were defined as “poor” quality.

### Statistical analysis

To determine inter-rater reliability among the four reviewers, an intraclass correlation coefficient (ICC) was calculated for each domain using a two-way random effects model. This process was performed for each of the included CPGs. Agreement among reviewers was classified as being excellent (0.81–1.00), good (0.61–0.80), moderate (0.41–0.60), fair (0.21–0.40), or poor (<0.20) based on calculated ICC value. Statistical analyses were performed in Stata 13 and reported with 95% confidence intervals. A two-tailed p value of 0.05 was considered statistically significant.

## Results

### Literature search

**Fig. 1:**
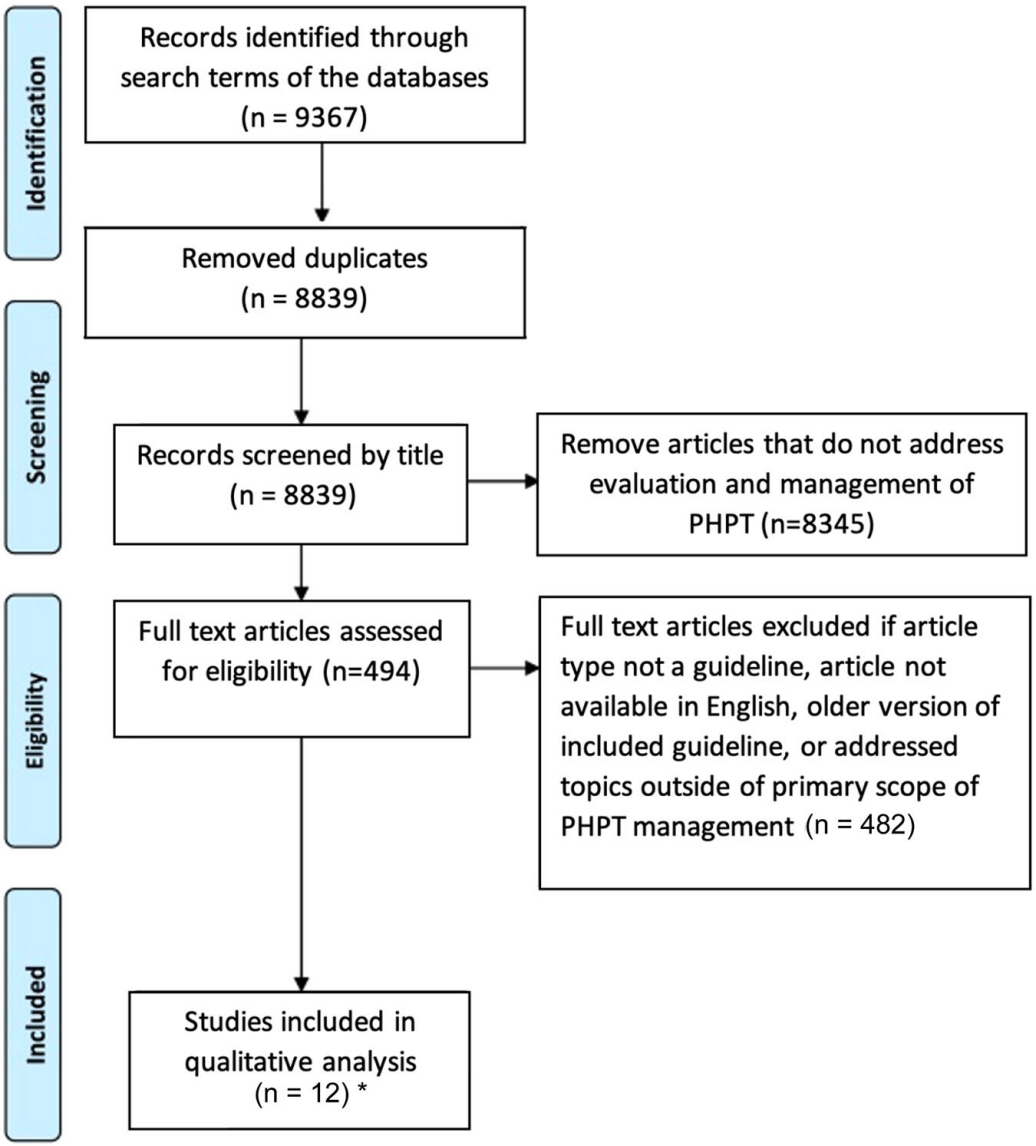
Preferred reporting items for systematic reviews and meta-analyses (PRISMA) flow diagram demonstrating literature search process and final guideline selection. PHPT primary hyperparathyroidism. *Two of the ten articles were available in two parts (4th international workshop guidelines and ANZBMS guidelines). These two-part guidelines were treated as a single guideline for the purposes of this AGREE II appraisal

Articles were reviewed in a systematic process (Fig. 1). Among the twelve guidelines meeting inclusion criteria, there were multiple published as two-part series (ANZBMS, Australia and New Zealand Bone and Mineral Society, and 4th International Conference). For the purposes of the analysis, ratings across the six domains were performed based on the combined two-part CPG series. Included guidelines are summarized in Table 2, along with the author, year of publication, abbreviation used in this analysis, funding sources, review methods utilized in development, and basic information included in the guideline.

**Table 2:** Summary of guideline characteristics

First author, year	Development group	Region of origin	Funding	Evidence base	Guideline content
Kukora, 2005	American Association of Clinical Endocrinologists (AACE) and The American Association of Endocrine Surgeons (AAES)	USA	None	Literature review and formulation of recommendations	Position statement on diagnosis and management
Zini, [25]	Italian Association of Clinical Endocrinologists (AME)	Italy	None	Literature review and formulation of recommendations based on Grading of Recommendations, Assessment,Development, and Evaluation (GRADE) system	Position statement on diagnosis, evaluation and management
Bandeira, 2013	Brazilian Society for Endocrinology & Metabolism (BSEM)	Brazil	None	Systematic literature review and formulation of recommendations based on Oxford Center for Evidence-Based Medicine grading system	Position statement on diagnosis, manifestations, and management
Rosato, [20]	Italian Association of Endocrine Surgery Units (UEC)	Italy	None	Consensus based on panel of experts formulated virtually and at an National Congress	Consensus statement on diagnosis and management
Udelsman and Bilezikian, [14, 22]	Fourth International Workshop (4th Inter)	International	None	Literature review and formulation of guidelines by workshop and expert panels	Consensus statement on diagnosis and surgical management
Marcocci, [17]	Società Italiana di Endocrinologia, SIE	Italy	None	Literature review and formulation of recommendations based on Grading of Recommendations, Assessment,Development, and Evaluation (GRADE) system	Questions and answers in the form of recommendations for diagnosis and management
Khan, 2016	Canadian Endocrine Update, McMaster University, and Western University (Canada)	Canada	Calcium Disorders Clinic, McMaster University Hamilton, Canada	Systematic literature review and formulation of recommendations	Review and recommendations on evaluation, diagnosis and management
Wilhelm, [24]	American Association of Endocrine Surgeons (AAES)	USA	None	Systematic literature review and formulation of recommendations by experts using American College of Physicians grading system for evidence-based guidelines	Position statement on diagnosis and management
NICE, 2019	National Institute for Health and Care Excellence (NICE)	UK	NICE and Royal College of Physicians	Literature review and interpretation of evidence	Guideline on diagnosis and management
Milat and Miller, [18, 19]	Endocrine Society of Australia, The Australian & New Zealand Endocrine Surgeons, and The Australian & New Zealand Bone and Mineral Society (ANZBMS)	Australia and New Zealand	None	Systematic literature review based on the systematic approach for adaptation of guidelines (ADAPTE) and formulation of recommendations	Position statement on diagnosis, management, and post-operative follow-up
Bilezikian, [38]	International Group of Endocrinologists (IGE)	International	Amolyt, Ascendis, Calcilytix, and Takeda Pharmaceutical Company	Systematic literature review and opinion-based recommendations from >50 experts and GRADE system	Consensus statement on diagnosis and management
Bollerslev, [39]	European Society of Endocrinology (ESE)	Europe	Takeda Pharmaceutical Company and Ascendis Pharma	Systematic literature review and expert consensus	Consensus statement on diagnosis and management

### Guideline appraisal

Using the scaled domain scores, the CPGs were evaluated based on the AGREE II criteria, with results shown in Table 3. Only one CPG, the AAES guideline, was found to be “high” quality, meeting the requirement of scaled domain scores >60% across five or more domains. The average scaled score across all six domains for this AAES CPG was 74.6 ± 31.4%. The CPGs produced by ANZBMS and European Society of Endocrinology (ESE) were “average” quality, with four domains meeting the >60% requirement. The remaining CPGs were characterized as “low” quality as they did not achieve the scaled score of >60% on at least three domains. Overall, the highest scoring domain across all CPGs was “clarity of presentation,” with an average score of 60.5%, followed by “scope and purpose” with an average score of 56.0%. The lowest scoring domain across all CPGs was “applicability” with an average score of 19.8%.

**Table 3:** Scaled domain scores justifying guideline quality interpretation

Guideline	Domain 1	Domain 2	Domain 3	Domain 4	Domain 5	Domain 6	Overall	Interpretation
	Scope and purpose (%)	Stakeholder involvement (%)	Rigor of development (%)	Clarity of presentation (%)	Applicability (%)	Editorial independence (%)	Mean scaled score	Quality
AACE/ AAES	12.5	8.3	13.6	27.8	1.0	0.0	10.5	Low
AME	37.5	37.5	23.5	75.0	9.4	50.0	38.8	Low
BSEM	38.9	33.3	26.6	43.0	7.3	50.0	33.2	Low
UEC	33.3	41.7	8.3	19.5	6.9	50.0	26.6	Low
4th Inter	69.5	51.4	28.2	61.1	9.4	0.0	36.6	Low
SIE	48.6	23.6	19.4	69.5	11.1	50.0	37.0	Low
Canada	77.8	54.2	54.8	58.3	11.5	52.1	51.5	Low
AAES	83.4	68.0	75.0	100.0	15.3	100.0	73.6	**High**
NICE	54.2	27.8	25.0	34.7	54.2	0.0	32.7	Low
ANZBMS	83.3	79.2	79.2	97.3	38.8	50.0	71.3	Average
IGE	49.2	71.5	66.3	52.9	19.9	50.0	51.6	Low
ESE	83.3	78.8	79.2	87.3	52.2	50.0	71.8	Average
Mean ± SD	56.0 ± 23.4	47.9 ± 23.1	41.6 ± 27.2	60.5 ± 26.5	19.8 ± 18.2	41.8 ± 28.9	-	-

4th International Workshop on the Management of Asymptomatic Primary Hyperparathyroidism in Florence, Italy (2013), *AACE/AAES* American Association of Clinical Endocrinologists and The American Association of Endocrine Surgeons, *AME* Italian Association of Clinical Endocrinologists, *BSEM* Brazilian Society for Endocrinology & Metabolism, *SIE* Società Italiana di Endocrinologia, *ANZBMS* The Australian & New Zealand Bone and Mineral Society, *NICE* National Institute for Health and Care Excellence, *AAES* The American Association of Endocrine Surgeons, *UEC CLUB* Italian Association of Endocrine Surgery Units

### Interrater reliability

ICC was calculated for each domain to determine interrater reliability (Table 4). Across all six domains, excellent (>0.80) interrater reliability was achieved. Domain 6, “Editorial Independence”, achieved the best agreement with an ICC of 1.00 (100.0%). Although still considered “excellent” with an ICC of 0.89 (89.0%), the domain with the poorest agreement was Domain 2, stakeholder involvement. This domain also contained the largest confidence interval, with the 95% confidence interval spanning from 0.84 to 0.94.

**Table 4:** Inter-rater reliability assessment for each AGREE II domain

Agree II domain	Intraclass correlation coefficient (ICC)	95% confidence interval
Domain 1: Scope and purpose	0.96	[0.93–0.99]
Domain 2: Stakeholder involvement	0.89	[0.84–0.94]
Domain 3: Rigor of development	0.93	[0.89–0.97]
Domain 4: Clarity of presentation	0.94	[0.92–0.96]
Domain 5: Applicability	0.96	[0.93–0.99]
Domain 6: Editorial independence	1.00	[1.00–1.00]

*AGREE II* Appraisal of Guidelines for Research and Evaluation Instrument, *ICC* intraclass correlation coefficient

## Discussion

While surgical intervention remains the standard of care for most patients diagnosed with PHPT, the availability of novel medical treatments and wide array of diagnostic modalities has led to a recent evolution in management. This report systematically assesses the quality of twelve CPGs detailing various elements of PHPT diagnosis and treatment. Guidelines were produced by working groups from several individual countries, as well as one guideline produced through an international collaboration (Udelsman et al.) [22]. A majority of guidelines were comprehensive in scope, detailing typical patient presentation, epidemiology, diagnostic measures, preoperative planning, surgical details, medical approaches to treatment, and patient follow-up/long-term evaluation. Despite these advantageous features, the AGREE II instrument elucidated several significant methodological, presentation-related, and applicability limitations in existing guidelines. Highlighting these potential areas for improvement holds the potential to facilitate the improvement of future guidelines.

The AGREE II is a novel guideline appraisal system for categorizing quality on the basis of six domains: scope and purpose, stakeholder involvement, rigor of development, clarity of presentation, applicability, and editorial independence. In this study, we found “clarity of presentation” to be highest scoring domain across all included articles for evaluation and management of PHPT. The strength of this domain is relevant for managing PHPT, as prior studies have stressed the importance of using systems that increase clarity of guidelines in order for them to be useful in managing endocrine conditions [27]. “Scope and purpose” was the second strongest-scoring domain on average, which is important characteristic given that hyperparathyroidism can manifest in a multitude of manners, presenting with or without symptoms as well as with or without hypercalcemia, in addition to varying etiologies of primary versus secondary or tertiary hyperparathyroidism [28].

The two lowest scoring domains, “applicability,” and “rigor of development”, however, are equally important and relevant in the development of guidelines addressing the management of PHPT. The “rigor of development” domain includes assessment of guidelines based on inclusion of systematic methods of development, clear descriptions of the reasoning behind recommendations with direct links to evidence-based medicine supporting literature, expert review, and procedures for updating the guideline. These points are particularly relevant to management of PHPT, as incidence and surgical intervention rates have fluctuated over time due to underlying etiologic factors as well as the advent of new technologies, such as automated serum calcium testing [29]. Therefore, it is essential for CPGs to be subject to high rigor of development to keep pace with the constantly evolving literature and incidence of disease. Moreover, “applicability” is also vital for longevity of a guideline and for ensuring that the CPG is instituted in clinical practice, which historically has been proven difficult in PHPT management [30–32]. Future guidelines should aim to increase applicability by including discussions on facilitators and barriers to guideline implementation, advice and tools on how to implement recommendations in clinical practice, discussion of resource implications in applying the guideline, and monitoring/auditing criteria. These improvements would enhance use of CPGs in clinical practice and enable feedback on performance of each guideline after its creation.

There was a consistent lack of quality across CPGs for diagnosis and treatment of PHPT. The only guideline deemed to be ‘high’ quality (attaining a scaled domain score of >60% in five or more quality areas) was produced by the American Association of Endocrine Surgeons (AAES) in 2016, with an average scaled domain score of 73.6%. This CPG scored highly in the domains of “clarity of presentation” and “editorial independence.” This suggests that the recommendations clearly delineated management objectives to optimize usability in a clinical context and that relevant conflicts of interest were properly addressed to minimizing influence of bias or competing interests. The lowest-scoring domain for the AAES guideline was in “applicability”, suggesting potential barriers to applying guideline recommendations were insufficiently addressed, no additional tools were provided to overcome identified limitations, and that processes were not described for regular auditing and updating of the CPG. This applicability domain was also identified as having the lowest average quality across all guidelines surveyed (16.5%). Addressing this shortcoming would require future guidelines to discuss guideline implementation, resource utilization, and periodic updating. Furthermore, the next highest performing guideline produced by the ESE working group was “average” quality, meeting a threshold of >60% in only four domains. Given that all other remaining guidelines fell below the threshold of >60% in five or more quality areas, the authors recommend consultation of the 2016 AAES guideline due to its strong methodologic rigor. Nonetheless, the authors acknowledge that the evidence base used to support clinical recommendations in other published guidelines may be highly accurate and reliable despite the less detailed description of their developmental methodology.

### Summary of clinical recommendations

There were several universal recommendations identified within guidelines addressing the diagnosis and treatment of PHPT. Diagnostic testing considerations for patients presenting with symptoms suggestive of PHPT included serum calcium and phosphate levels, 25-hydroxyvitamin D levels, 24-hour urine collection, intact parathyroid hormone measurement, kidney function (creatinine), and bone density via DEXA scan. A majority of guidelines specified that surgery was indicated for all patients under the age of 50 presenting with biochemically-confirmed PHPT. Additional indications for surgery include T-score < −2.5 measured at the lumbar spine, hip, femoral neck, or distal radius as well as fragility fractures caused by osteoporosis secondary to primary hyperparathyroidism. Signs of kidney dysfunction including decreased creatinine clearance <60 cc/min favors surgical intervention as well. Imaging should be used primarily for preoperative planning, with modalities such as cervical ultrasonography, computed tomography (CT), or Tc-99m (Sestamibi) scanning. Medical treatments aimed at reducing calcium levels, such as cinacalcet and bisphosphonates, may be useful for controlling calcium levels and providing skeletal protection in patients with contraindications to surgery. Finally, guidelines unanimously advocated for biochemical confirmation of cure, such as eucalcemia and normalization of PTH at postoperative follow-up.

### Limitations

This study had several limitations. The framework described in the AGREE II assessment method emphasizes methodologic rigor of the guideline and does not necessarily assess the evidence base used to support the clinical recommendations. This study serves to highlight which guidelines were developed comprehensively by addressing each of the domains assessed by the AGREE II instrument. Further studies are needed to address how each of these recommendations performs in clinical practice. Moreover, the subjective nature of rating guidelines could introduce bias. We sought to minimize this effect by recruiting certified, independent guideline reviewers with an established track-record of adhering to established AGREE II methodology. High ICCs achieved across all domains suggest that rating biases were ultimately inconsequential. It should be disclosed that our group is composed of endocrine surgeons with a background in Otolaryngology – Head & Neck Surgery. This background could cause unconscious bias favoring guidelines that place greater emphasis on surgical considerations. Furthermore, it is possible that authors of the current article residing in the United States may subconsciously favor recommendations produced of the association presiding over their practice. This limitation could result in favorable AGREE II scoring for the American Association of Endocrine Surgeons guideline.

Importantly, parathyroid disorders manifest as a broad spectrum of disease. This study focused on primary hyperparathyroidism without addressing other causes of hyperparathyroidism, including familial hyperparathyroidism, secondary and tertiary hyperparathyroidism, or hyperparathyroidism secondary to systemic disease. Furthermore, we excluded articles that may offer valuable information for management of PHPT but did not feature specific clinical guidelines, such as review articles or meta-analyses. It is important to consider other sources of information in clinical practice to address the shortcomings of the guidelines included in this study. Despite these limitations, insights generated herein represent a valuable addition to the emerging literature that critically evaluates the methodological quality of guideline development in endocrinology [33–37]. Lastly, guidelines receiving financial support from industry partners were authored by the International Group of Endocrinologists (IGE)^38^ and European Society of Endocrinology (ESE) [39]. Whether this funding influenced guideline content remains a matter of speculation.
